# Hirschsprung’s disease presenting as intractable anemia: a report of two cases and review of the literature

**DOI:** 10.1186/s12887-020-02423-z

**Published:** 2020-11-16

**Authors:** Xiaoang Sun, Jun Chu, Chenchen Li, Zhaohui Deng

**Affiliations:** 1 Department of Gastroenterology, Shanghai Children’s Medical Center, Shanghai Jiaotong University School of Medicine, Shanghai, 200127 PR China; 2 Department of General Surgery, Shanghai Children’s Medical Center, Shanghai Jiaotong University School of Medicine, Shanghai, 200127 PR China; 3 Department of Gastroenterology, Shanghai Children’s Medical Center, Shanghai Jiaotong University School of Medicine, 1678 Dongfang Road, Pudong New District, Shanghai, 200127 PR China

**Keywords:** Anemia, Hirschsprung’s disease, Hemoglobin

## Abstract

**Background:**

This report summarizes the clinical characteristics of intractable anemia as part of the clinical presentation of Hirschsprung’s disease (HD) and aims to strengthen clinicians’ ability to recognize early signs of HD.

**Case presentation:**

An 11-year-old boy with a 6-year history of intractable anemia, low hemoglobin level (55 g/L), poor response to oral iron supplementation and blood transfusion, and difficulty with defecation was diagnosed with HD. A 19-month-old boy with a 3-month history of intractable anemia, low hemoglobin level (64 g/L), poor response to oral iron supplementation and blood transfusion, delayed meconium passage, and history of intestinal obstruction was also diagnosed with HD. Both patients underwent surgery, after which anemia was corrected effectively in both cases. Two more cases of intractable anemia as the chief complaint and diagnoses of HD over different durations since the onset of anemia (ranging from 1.7 years to 21 years) were identified in a literature search. Both patients underwent surgery, after which anemia was corrected.

**Conclusions:**

Intractable anemia as part of the clinical presentation of HD is extremely rare. Detailed inquiries of medical histories and physical examinations are key to early diagnoses and preventing misdiagnoses. Anemia in HD patients may primarily be caused by impaired iron absorption due to HD.

## Background

Hirschsprung’s disease (HD), also known as congenital megacolon, is an absence of the enteric neurons. It is a common gastrointestinal malformation in pediatrics, with an incidence of 1 per 5000 newborns [1]. Most patients with HD are diagnosed during the neonatal period due to intestinal obstructions. However, part of HD with atypical symptoms (mostly differing degrees of constipation) in china may be undiagnosed or misdiagnosed, resulting in delayed treatment. Intractable anemia as part of the clinical manifestations of HD is very rare. In this study, we report about two patients with HD with intractable anemia as the chief complaint who were treated at the Shanghai Children’s Medical Center. Additionally, we review the literature and summarize the clinical features of intractable anemia as part of the clinical manifestations of HD.

## Case presentations

Before accessing the patients’ records, we obtained informed consent from the patients’ parents to include their child in the study and publish the results.

Here, we report about two patients with confirmed HD and intractable anemia as the chief complaint who presented at the Shanghai Children’s Medical Center between April 25, 2019 and June 30, 2020.

**Fig. 1:**
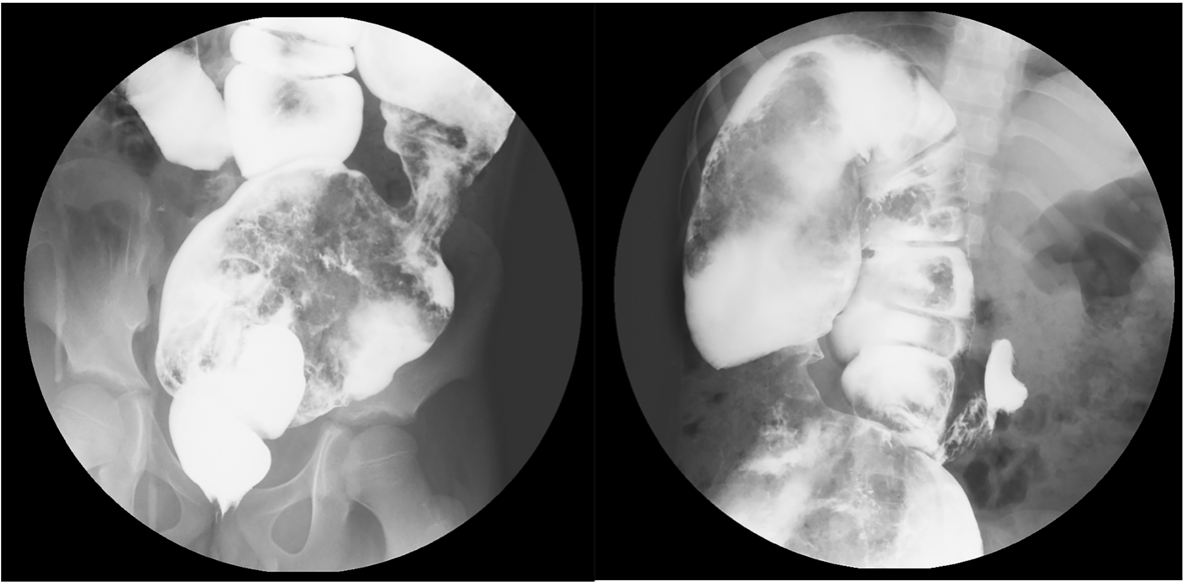
A barium enema examination revealed that, after injection of contrast agent into the anus, combined with fluoroscopy, the rectum was narrow, and the upper intestine segment was significantly dilated

An 11-year-old boy (case 1) was admitted to our hospital with the chief complaint of “a pale complexion for the last 6 years which has worsened over the last 1 month.” Born in the countryside, the boy had a history of constipation, but his family did not pay attention to it and did not take him to a doctor until his complexion turned pale at 5 years of age. The child was taken a local hospital with the chief complaint of anemia, but the doctor did not inquire about the child’s past medical history in details. A gastrointestinal endoscopy was performed, and eosinophilic gastroenteritis was suggested, therefore, no HD-related examinations were conducted. He was treated with an iron supplement (ferrous succinate tablets; Jinling Pharmaceutical Company Limited, Nanjing Jinling Pharmaceutical Factory) and prednisone (Tianjin Lisheng Pharmaceutical Co., Ltd.). However, anemia persisted. When the child was admitted to our hospital, we asked for his medical history and learned that he had a poor appetite, difficulty with defecation, and less than the normal amount of dry stool. The time required for complete meconium passage was unknown. A physical examination revealed a height of 137 cm, a weight of 27.8 kg, and a body mass index (BMI) of 14.81 kg/m^2^. He also appeared anemic, and he had slightly pale skin and a soft abdomen without obvious distention. Laboratory tests showed a hemoglobin (Hb) level of 55 g/L, a mean corpuscular volume (MCV) level of 60.5 fL, a mean erythrocyte Hb content (MCH) level of 15.3 pg, and a mean erythrocyte Hb concentration (MCHC) level of 252 g/l. The blood smear showed visible evidence that the red blood cells were of different sizes and were lightly stained. The bone marrow image showed significantly active proliferation, erythroid hyperplasia, and the disappearance of extracellular iron. An erythrocyte osmotic fragility test, folate and vitamin B12 determination, and Coomb’s test were all negative. His serum iron (SI) (7.45 mmol/L) and ferritin (1.1 ng/ml) were low, suggesting iron deficiency anemia, and his 25-hydroxy vitamin D (4.89 ng/ml), albumin (ALB) (30 g/L) were also low. But his lectrolytes were normal. An enhanced pelvic computed tomographic examination revealed a significantly dilated colon with colonic wall thickening. A barium enema (BE) examination suggested HD (Fig. 1). The pathological examination of a full thickness rectal biopsy revealed hypertrophic nerve plexuses without ganglion cells in the colonic submucosa. Calretinin staining was negative, confirming the diagnosis of HD. Under general anesthesia, the patient underwent laparoscopic rectosigmoid pull-through according to Soave. A total resection of a bowel segment about 30 cm long and an aganglionotic segment about 10 cm long was performed. A colorectal anastomosis was performed. A postoperative anal expansion was performed with an anal dilator. He recovered well after the Soave surgery. He was followed up for 1 year with a routine blood test every 3 months, and his hemoglobin level was maintained at above 110 g/L. After 1 year, his height, weight and BMI were 150.4 cm, 37 kg and 16.36 kg/m^2^, respectively.

A 19-month-old boy (case 2) was hospitalized with the chief complaint of a “pale complexion for 3 months.” He had been to the local hospital for an intestinal obstruction and iron deficiency anemia. His symptoms improved after receiving conservative treatments, (fasting, fluid rehydration, and iron supplementation). Therefore, the doctor did not pay attention to the child’s past medical history or conduct a BE examination; however, the anemia later returned, and the child was brought to our hospital. We learned that he had a history of delayed meconium passage and intestinal obstruction. The patient had difficulty defecating at normal times, with less than normal amounts of stool. His height and weight were 79 cm, 10 kg, and his BMI was 16.02 kg/m^2^, and he had considerable abdominal distention. Laboratory tests results were as follows: Hb (64 g/L), MCV (75.2 fL), MCH (20.7 pg), MCHC (276 g/L); SI (6.47 mmol/L), ferritin (13.7 ng/ml). An examination of his cell morphology showed cells moderately different in size and obviously lightly stained. His bone marrow cytology was markedly active and there was a lack of extracellular iron. His ALB (31.6 g/L) and 25-hydroxy vitamin D (6.34 ng/ml) levels were low, but his electrolyte, folic acid, and vitamin B12 levels were normal. A BE examination indicated HD. A full thickness rectal biopsy revealed that there were no ganglion cells in the colonic submucosa, confirming the diagnosis of HD. Under general anesthesia, the patient underwent laparoscopic.

rectosigmoid pull-through according to Soave. During the surgery, we found that the length of the aganglionotic bowel segment was 8 cm and the length of the resected intestinal segment was 15 cm. Calretinin staining of the aganglionotic segment was negative. A colorectal anastomosis was performed after the resection was completed. He recovered well after the Soave surgery. He was followed up for 3 month, and his routine blood test results revealed that his hemoglobin level was maintained at above 100 g/L. Now, his height, weight and BMI were 83 cm, 11.6 kg and 16.84 kg/m^2^, respectively.

## Discussion and conclusion

HD is a common gastrointestinal malformation in pediatric patients and a common cause of neonatal intestinal obstructions. HD is characterized by the absence of ganglion cells in the submucosal and myenteric plexuses of the distal bowel wall, which leads to persistent spasming and narrowing of the affected bowel segment, causing intestinal obstruction [2]. The most prominent symptoms of HD are vomiting, abdominal distension, and defecation dysfunction during early life. Intractable anemia as part of the manifestation of HD is very rare.

A search of the literature published before 2020 was conducted using the China National Knowledge Infrastructure and Wanfang Data with “anemia” and “congenital megacolon” as keywords, and one article was retrieved [3]. A similar search in PubMed retrieved another article published before 2020 [4]. We reviewed these four cases and have summarized their clinical characteristics. The BMIs of the two children treated in our hospital were 14.81 kg/m^2^ (case 1) and 16.02 kg/m^2^ (case 2), which indicates that both children had lean bodies, and the vitamin D and albumin values for both children were lower than normal. The two children from the cases in the literature showed stunting or malnutrition. The HD courses in these four cases were relatively long, ranging from 1.7 years to 21 years. All four patients presented to the hospital with primary symptoms of intractable anemia, accompanied by the clinical manifestation of difficulty with defecation and irrelevant abdominal signs. Only one patient had abdominal distension. The durations of the iron deficiency anemia in all of these cases were long, ranging between 3 months and 6 years. The iron deficiency anemia did not respond well to iron supplementation and blood transfusions, but it was effectively corrected with megacolonectomies (Table 1).

**Table 1:** Clinical characteristics of patients with congenital megacolon included in this study

Case	Sex	Nutritional status	Age at diagnosis (Years)	Duration of anemia (Years)	Main manifestation	Gastrointestinal (GI) symptoms	Hemoglobin level (HB)	Type of anemia	Treatment history	Treatment method	Follow-up results
1	Male	BMI 14.81 kg/m^2^ Vitamin D deficiency, Hypoproteinemia	11	6	Pale complexion	Difficulty with defecation	55 g/L	Iron deficiency anemia	Iron supplement/ prednisone/blood transfusion	Surgery	Anemia corrected and constipation resolved
2	Male	BMI 16.02 kg/m^2^ Hypoproteinemia, Vitamin D deficiency	1.7	0.3	Pale complexion	Constipation and bloating	64 g/L	Iron deficiency anemia	Iron supplement/blood transfusion	Surgery	Anemia corrected and abdominal distension improved
3	Male	Malnutrition, emaciation	5	4.5	Pale complexion and lack of strength	Difficulty with defecation with little amount of stool	52 g/L	Iron deficiency anemia	Iron supplement/blood transfusion	Surgery	Anemia corrected and normal bowel movement
4	Male	Malnutrition	21	Several months	Fatigue	Difficulty with defecation	48 g/L	Iron deficiency anemia	Iron supplement/blood transfusion	Surgery	Anemia corrected and normal bowel movement

Because of malnourishment caused by chronic obstructions associated with HD, which indirectly or directly affects the digestion and absorption of nutrients (including iron and other blood-forming materials), children with HD often experience varying nutrient deficiencies, significant growth retardation, and nutritional anemia. The two children treated in our hospital had low BMI values, vitamin D deficiency, and hypoproteinemia. The authors of the two papers from our literature search did not provide BMI values of their patients; however, they did mention that the children had poor appetites, square skulls, weight loss, malnutrition, and fatigue. Therefore, besides paying attention to anemia in children with HD, it is necessary to assess the overall nutritional status of the patients.

The four patients in this report (two from our hospital and two from the literature searches) were diagnosed with HD at older ages (11, 1.7, 5, and 21 years, respectively). Among the four patients, anemia may arise after delayed diagnose of HD; two children in the literature, one case did not defecate meconium 4 days after birth, the local doctor only examined a plain abdominal radiograph and used anal dilation and laxative treatment. As the child developed symptoms of anemia later in life, the doctor only managed it symptomatically and did not perform BE or other examinations that would have aided in the diagnosis of HD. The specific diagnosis and treatment for the 21-year-old patient were not provided in the report, but it was reported that the individual had a history of depression and constipation. We speculated that because the patient had problems with depression, family members and doctors did not pay attention to the constipation symptoms, and this led to a delayed HD diagnosis. It appears that the primary reason for the delay in the diagnosis of these four individuals was that the initial symptoms for each patient were mild and did not get the attention of the family. In addition, the patients initially visited the doctor with the main complaint of intractable anemia, prompting the clinicians to focus on that rather than inquire in detail about any past medical history. Meanwhile, these patients had an atypical gastrointestinal presentation that remained unexplored. In these four cases, only the infant had a definitive history of defecation dysfunction and abdominal distention. The other three patients who were older children or in early adulthood had no significant abdominal signs, and their gastrointestinal presentation was only difficulty with defecation. With constipation as a gastrointestinal presentation in older children, more than 90% of the cases are functional in nature [5], and it is uncommon to associate constipation with intractable anemia. Thus, a diagnosis of HD can be missed. Therefore, HD is not frequently recognized early, and it is not diagnosed and treated promptly by doctors, ultimately leading to intractable anemia. It is thus critical that clinicians strive to identify the cause of anemia in patients, without simply treating it with iron supplementation alone. This is particularly true for children with constipation, where clinicians should conduct a physical examination and take a careful and comprehensive look at the patient’s medical history, which may open up the possibility of HD sooner.

All four patients had moderate to severe iron deficiency anemia, which was mainly caused by impaired absorption, and utilization of iron and the loss of iron or blood because of undiagnosed and untreated HD. Traditional iron supplements, and even blood transfusions, had little or no effect on the anemia in any of the four patients. In these cases, treating the primary disease and surgically removing the affected bowel segments were the only effective approaches to improving the anemia. Traditionally, a majority of the iron and other nutrients are absorbed in the small intestine, and consequently, iron deficiency anemia is less likely to occur in a patient with HD. However, research has shown that iron transporter genes, such as DCYTB, DMT1, and FPN1, are expressed in the colon tissue and regulated by hepcidin. Therefore, the colon also partially participates in iron absorption. Hepcidin is a recently identified hormone-like protein synthesized and secreted by the liver. It regulates intestinal iron absorption and the body’s iron storage by inhibiting iron absorption by enterocytes and iron release from reticuloendothelial cells. Mutations in ferroportin (e.g., N144D/T/H, Y64N, C326S/Y/X, A69T, D181V, A77D, and SLC40A1) [6–8] can prevent interactions between hepcidin and ferroportin, thus inducing the internalization or degradation of ferroportin, inhibiting the release of iron into the plasma, and reducing iron absorption [6–8].

Moreover, anemia in patients with HD may be associated with altered intestinal microenvironments. A recent study revealed that it lacks a part of probiotics in the intestines of children with HD, the intestinal microecology balance is broken, and the biological barrier provided by the intestinal mucosa is damaged [9]. The microbes in the colon not only affect the solubility and forms of trace minerals considerably [9] but also regulate the expression of iron transporter genes. Therefore, the composition and quantity of the intestinal microbiota affect iron absorption [10]. Other components within the intestinal microbiota also stimulate expression of hepcidin, which inhibits the release of iron into plasma from macrophages and small intestinal enterocytes [11].

Chronic intestinal inflammation not only impairs iron absorption but also leads to a loss of iron or blood, thus aggravating anemia. Idiopathic chronic inflammation is common in patients with HD, and proteinase-activated receptor-1 (PAR-1) and proteinase-activated receptor-2 (PAR-2) are expressed in children with HD [12]. Par-1 and Par-2 play an essential role in the development of intestinal inflammation by promoting the release of pro-inflammatory cytokines (IL-6 and IL-8) and chemokines (CXCL1 and CXCL8), consequently aggravating iron deficiency [12]. Additionally, interleukin 6 (IL-6) upregulates the expression of the hepcidin gene [11, 13], and elevated IL-6 levels are closely associated with increased transferrin receptor density [14].

Anemia as part of the clinical presentation of HD is very rare, and its mechanism necessitates further investigation. In clinical practice, it is important to obtain a detailed medical history and conduct thorough physical examinations to avoid missed diagnoses or misdiagnoses. In this report, we summarized the clinical characteristics of this type of HD, with an aim to provide additional information for clinicians to use in their practice. HD-associated anemia responds poorly to routine anemia treatments. The key is to treat the primary disease, which may be HD. Therefore, early diagnosis and intervention are critical for the improvement of the quality of life in pediatric patients with HD. We believe this report can provide clinicians with information that will help them diagnose HD earlier and also reduce the occurrence of missed diagnoses and misdiagnoses.

## Data Availability

The datasets used and/or analyzed during the current study are available from the corresponding author upon reasonable request.

## Abbreviations

HD: Hirschsprung’s disease
BE: Barium enema
CNKI: China National Knowledge Infrastructure
PAR-1: Proteinase-activated receptor-1
PAR-2: Proteinase-activated receptor-2
IL-6: Interleukin 6
